# Novel Metabolites Genetically Linked to Salt Sensitivity of Blood Pressure: Evidence from mGWAS in Chinese Population

**DOI:** 10.3390/ijms26104538

**Published:** 2025-05-09

**Authors:** Xiaojun Yang, Bowen Zhang, Fuyuan Wen, Han Qi, Fengxu Zhang, Yunyi Xie, Wenjuan Peng, Boya Li, Aibin Qu, Xinyue Yao, Ling Zhang

**Affiliations:** Department of Epidemiology and Health Statistics, School of Public Health, Capital Medical University and Beijing Key Laboratory of Environment and Aging, Beijing 100069, China

**Keywords:** metabolomics, metabolome genome-wide association study, salt sensitivity of blood pressure, hypertension, biomarkers

## Abstract

This study aims to identify genetically influenced metabolites (GIMs) associated with SSBP and elucidate their regulatory pathways through metabolome genome-wide association studies (mGWASs). Untargeted metabolomics and genome-wide genotyping were performed on 54 participants from the Systematic Epidemiological Study of Salt Sensitivity (EpiSS). The mGWAS was conducted on 970 plasma metabolites, and their potential biological mechanisms were explored. The multivariable logistic regression model and mendelian randomization (MR) were employed to investigate the association and causal relationship between GIMs and SSBP. Metabolomic analysis was performed on 100 subjects in the replication analysis to validate the GIMs identified in the discovery set and their causal association with SSBP. The mGWAS revealed associations between 1485 loci and 18 metabolites. After performing linkage disequilibrium analysis, 368 independent mQTLs were identified and annotated to 141 genes. These functional genes were primarily implicated in the signal transduction of sinoatrial node and atrial cardiac muscle cells. Five key genes were identified using CytoHubba, including *CAMK2A*, *TIAM1*, *RYR2*, *RBFOX1*, and *NRXN3*. One-sample MR analysis revealed 14 GIMs with causal associations to SSBP, with LysoPC (0:0/22:5*n*-3) positively associated with SSBP (*p* < 0.05). The causal relationship between Phe-lle and SSBP was validated in the replication analysis. This study elucidates the genetic regulatory mechanisms underlying metabolites and identifies GIMs that are causally associated with SSBP. These findings provide insights into identifying metabolic biomarkers of SSBP and characterizing its genetic and metabolic regulation mechanisms.

## 1. Introduction

Salt sensitivity of blood pressure (SSBP), an intermediate genetic phenotype of hypertension, is characterized by distinct clinical features and pathophysiological changes [1]. Specifically, individuals whose blood pressure rises with salt loading and falls with salt depletion are classified as salt-sensitive (SS), while those without such changes are classified as salt-resistant (SR) [2]. SSBP is widely recognized as an independent risk factor for cardiovascular disease and death [3]. Over 50% of hypertensive patients exhibit SSBP, characterized by greater blood pressure fluctuations compared to normotensive individuals [4,5]. Previous studies have demonstrated that SSBP is influenced by both environmental and genetic factors, with heritability estimated to be as high as 74% in Black individuals [4,6]. Parksook et al. identified 18 genes and their corresponding SNPs associated with the pathogenesis of SSBP [6]. However, most candidate genes identified through genome-wide association studies (GWASs) are located in non-coding regions and exhibit small genetic effect sizes [7,8]. These mutations do not directly affect protein coding, which makes it challenging to elucidate the pathogenic mechanisms and regulatory pathways that connect genetic variations to diseases or phenotypes.

Compared to traditional single-omics approaches, metabolite genome-wide association studies (mGWASs) utilize metabolomics as an intermediate phenotype, providing deeper insights into gene functions and the metabolic regulatory mechanisms underlying diseases. mGWASs have emerged as a powerful tool for investigating the impact of genetic variations in metabolism and complex diseases [9]. Previous studies have demonstrated that most identified metabolic quantitative trait loci (mQTLs) not only exhibit catalytic and transport functions but also play a role in regulating gene expression [10,11]. Wang et al. conducted a large-scale mGWAS in patients with coronary artery disease (CAD), identifying seven novel mQTLs associated with CAD, and shedding light on the relationship between genetic variations and personalized metabolism in these patients [12]. Feofanova et al. performed a GWAS on 640 circulating metabolites, identifying 46 variant–metabolite pairs. Mendelian randomization (MR) analysis further identified genetically influenced metabolites (GIMs) associated with coronary heart disease (CHD), such as octadecanedioate, γ-CEHC, and γ-CEHC glucuronide [13]. Currently, mGWASs are being widely employed to identify genetic variation and investigate genetic regulatory metabolic pathways in complex diseases such as cardiovascular diseases [14], chronic kidney diseases [15], and asthma [16]. These studies provide valuable insights into the identification of early molecular biomarkers and the understanding of disease pathogenesis. However, the application of the mGWAS to unravel the molecular mechanisms underlying SSBP remains unexplored.

This study aims to perform an mGWAS on 970 plasma metabolites identified through untargeted metabolomics. Subsequent analyses of the identified mQTLs, including fine mapping, functional annotation, and enrichment analysis, aim to elucidate the underlying metabolic regulatory pathways and biological processes. Additionally, the causal relationships between GIMs and SSBP were evaluated through MR analysis, providing a robust scientific basis for the early identification of SSBP and the exploration of its genetic metabolic mechanisms.

## 2. Results

### 2.1. Basic Characteristics of the Participants

In the EpiSS study, 54 participants were included for both untargeted metabolomics and genotyping analyses. Among the participants, 30 individuals were classified as SS, while the remaining were classified as SR. Table 1 presents the basic characteristics of the study participants, who had a mean age of 57.57 ± 5.43 years, and of whom 17 (31.5%) were male. There were statistically significant differences in SBP, DBP, MAP, ΔMAP_1_, and ΔMAP_2_ between the SS and SR groups (*p* < 0.05).

### 2.2. Metabolite Genome-Wide Association Analysis

**Figure 1:**
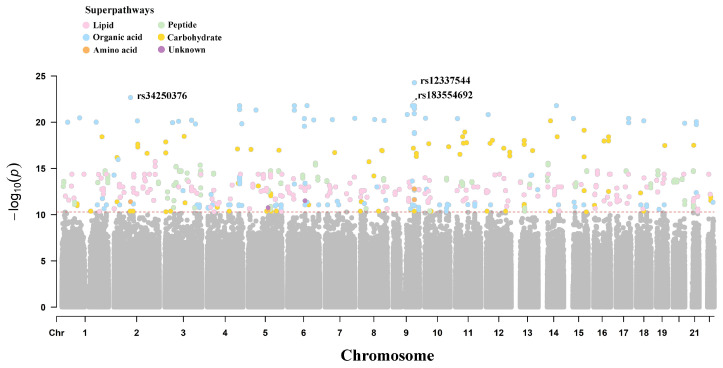
Manhattan plot of metabolic quantitative trait locus. The red dashed line indicates the Bonferroni threshold (*p* = 5.15 × 10^−11^). Colors in the legend represent metabolite superpathways, while gray dots represent SNPs not reaching genome-wide significance.

The 970 metabolites were categorized into eight superpathways: lipids, organic acids, amino acids, peptides, nucleotides, carbohydrates, cofactors and vitamins, and unknown, with 742 metabolites categorized as lipids. The GWAS was conducted on the levels of 970 metabolites, identifying 1616 variant–metabolite associations involving 1485 SNPs and 18 GIMs. These GIMs passed Bonferroni correction (*p* < 5 × 10^−8^/970 = 5.15 × 10^−11^). Figure 1 illustrates the results of the genome-wide association analysis for metabolites across the eight superpathways. Among the 18 GIMs, lipids were the most abundant, accounting for a total of 748 corresponding mQTLs (Supplementary Table S1). The top three most strongly associated mQTLs were all linked to feruloylquinic acid, with rs34250376 (*THNSL2*) and rs183554692 (*INVS*) playing a crucial role in visceral fat and nephronophthisis, respectively [17,18]. The mQTL with the most significant *p*-value, rs12337544, is located in the non-coding region.

### 2.3. Identification of Independent mQTLs

To further identify relatively independent association signals, linkage disequilibrium analysis was conducted on the tag SNPs. After clumping with a threshold of *r*^2^ < 0.02, 368 mQTL loci were retained. Table 2 presents the most significant mQTL loci for each metabolite. Among the 18 GIMs, 4 have been previously reported [19,20,21,22], including androsterone sulfate, LysoPC (0:0/14:0), 16α-hydroxy DHEA 3-sulfate isomer, and L-Cystine.

### 2.4. Effector Genes of Genetically Influenced Metabolites

**Figure 2:**
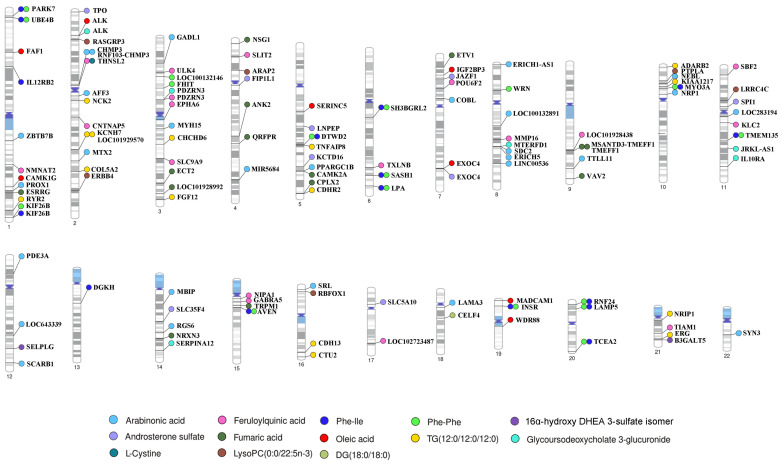
Genomic localization of independent mQTL annotated genes for 13 GIMs. The colored circles represent genetically influenced metabolites. The black horizontal lines on the chromosome indicate the positions of mQTLs, and the gene names annotated for the mQTLs are shown next to the circles.

Genetic variant loci and metabolites, as upstream and downstream components of genetic regulatory networks, provide limited insights into biological processes and pathogenic mechanisms. To identify the genes regulated by mQTLs, 368 tag SNPs were annotated using the reference genome (GRCh37), resulting in 141 genes corresponding to 13 metabolites. The mQTLs of the remaining five metabolites were not annotated to any genes. PhenoGram was employed to visualize the chromosomal locations of annotated genes (Figure 2). In addition, the GO annotations and KEGG pathways of these genes were analyzed to further explore the underlying biological processes (Supplementary Figure S1). The results indicated that these potential genes were primarily involved in biological processes such as sinoatrial node cell to atrial cardiac muscle cell signaling and sinoatrial node cell action potentials. Molecular functions were primarily related to transmembrane receptor protein tyrosine kinase activity and transmembrane receptor protein kinase activity and were associated with cellular components such as synaptic membranes. KEGG enrichment analysis revealed that potential genes associated with mQTLs were predominantly enriched in pathways such as proteoglycans in cancer, ovarian steroidogenesis, and motor protein functions.

### 2.5. Potential Proteins Related to Genetically Influenced Metabolites

**Figure 3:**
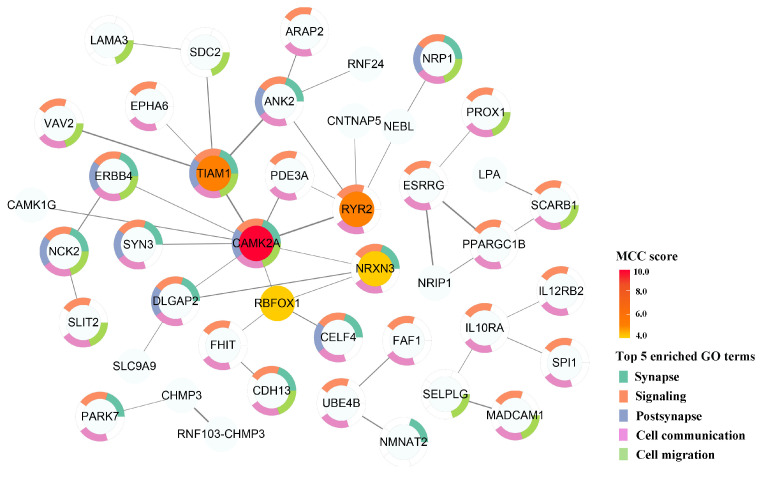
The PPI network of mQTL annotated genes. The top 5 key genes are filled with color, with redder nodes indicating higher maximal clique centrality (MCC) algorithm scores. The top 5 enriched GO terms are represented by five different colors.

A PPI network consisting of 43 nodes and 43 interacting edges was constructed from the STRING database based on the 141 annotated genes, excluding those that did not interact with the core network. These genes were predominantly enriched in the biological processes associated with signal transduction. The PPI network was visualized using Cytoscape 3.10.0 software and is shown in Figure 3. Five key genes in the PPI network were identified using the CytoHubba plug-in: *CAMK2A, TIAM1*, *RYR2*, *RBFOX1*, and *NRXN3*. Previous studies have suggested that these key genes play crucial roles in the physiological mechanisms underlying cardiovascular events, including atrial fibrillation [23], cardiomyopathy [24], and blood pressure regulation [25].

### 2.6. Associations Between GIMs and SSBP

Multivariable logistic regression analysis was conducted to evaluate the association between GIMs and SSBP (Supplementary Table S2). After adjusting for age, sex, and the first five principal components, the results showed that Phe-Ile and Phe-Phe were negatively associated with SSBP, with odds ratios (ORs) of 0.857 (95% CI: 0.742, 0.990) and 0.860 (95% CI: 0.748, 0.990), respectively. The other 16 GIMs showed no significant association with SSBP.

### 2.7. Mendelian Randomization Analyses

Based on the mGWAS results, one-sample MR analysis was conducted to further investigate the effects of variant–metabolite associations on SSBP. The simple and weighted PRSs at the individual level were calculated separately, and the 2SLSs method was employed to investigate the causal effect of metabolites on SSBP. The results revealed a potential causal relationship between 14 metabolites and SSBP, with lipid metabolites being the most predominant (Table 3). Thirteen metabolites, including arabinonic acid, feruloylquinic acid, Phe-lle, Phe-Phe, androsterone sulfate, fumaric acid, TGs (12:0/12:0/12:0), glycoursodeoxycholate 3-glucuronide, oleic acid, LysoPC (0:0/14:0), L-Cystine, X-MZ140RT42, and *N*-acetyl-l-aspartic acid, were negatively associated with SSBP (*p* < 0.05). In contrast, LysoPC (0:0/22:5*n*-3) was positively associated with SSBP (*p* < 0.05).

### 2.8. Construction of SNP–Gene–Metabolite Association Network Related to SSBP

**Figure 4:**
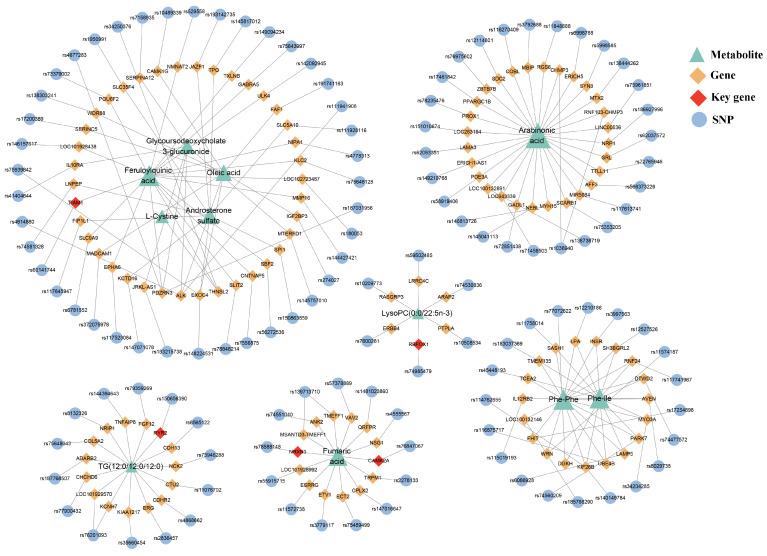
SNP–gene–metabolite association network related to SSBP. In this network, triangles, diamonds, and circles represent metabolites, genes, and SNPs, respectively. Red diamonds represent the five key genes identified by the CytoHubba plug-in. The larger the triangle, the greater the number of genes associated with the metabolite.

Among the 14 metabolites causally related to SSBP, the mQTLs of three metabolites were not annotated to genes. A network plot was constructed to visualize the SNP–gene–metabolite relationships for 11 metabolites associated with SSBP (Figure 4). The largest network subgraph was centered on five core GIMs: glycoursodeoxycholate 3-glucuronide, feruloylquinic acid, oleic acid, L-Cystine, and androsterone sulfate. The gene set associated with arabinonic acid was the largest, including 28 genes. Phe-Ile and Phe-Phe shared 14 common genes.

### 2.9. Replication Analyses

The baseline characteristics of the participants in the replication set are shown in Supplementary Table S3. Similarly to the discovery set, the differences in ΔMAP_1_ and ΔMAP_2_ between the two groups were statistically significant. However, no statistically significant differences were found in SBP, DBP, or MAP between the two groups in the replication set. Based on the results of the mGWAS analysis, further replication and validation were conducted in the population. The mGWAS analysis of nine metabolites (9 undetected) from 100 subjects (50 SS vs. 50 SR) revealed that oleic acid and Phe-lle were associated with nine and two mQTLs, respectively (*p* < 5 × 10^−8^) (Supplementary Table S4). The mQTLs of the remaining metabolites reached only the suggestive threshold (*p* < 1 × 10^−5^). The results of the one-sample MR analysis demonstrated a causal relationship between the weighted PRS of Phe-lle and SSBP (*p* < 0.05), suggesting a robust association between Phe-lle and SSBP (Supplementary Table S5).

## 3. Discussion

This study conducted an mGWAS analysis of 970 plasma metabolites from 54 participants in northern China, identifying 1616 variant–metabolite associations corresponding to 1485 mQTLs linked to 18 metabolites. After tag SNP screening, LD analysis, and fine mapping, 368 mQTLs associated with 18 metabolites were identified, corresponding to 141 genes. These functional genes, potentially regulating metabolites, were primarily involved in the composition of cell signaling molecules and signal transduction in sinoatrial node cells. Five key genes (*CAMK2A*, *TIAM1*, *RYR2*, *RBFOX1*, and *NRXN3*) were identified using the CytoHubba plug-in and were found to play a crucial role in the physiological and pathological mechanisms underlying cardiovascular events. In the one-sample MR analysis, 18 genetically influenced metabolites identified through the mGWAS were used to conduct a causal association analysis with SSBP, revealing that 14 metabolites had a causal relationship with SSBP. LysoPC (0:0/22:5*n*-3) was identified as a risk factor for SSBP, while the other 13 metabolites were identified as protective factors. The replication analyses revealed that oleic acid and Phe-lle were significantly associated with nine and two mQTLs, respectively, and Phe-lle remained causally associated with SSBP.

mQTLs identified through mGWASs are directly involved in metabolite regulation and exhibit larger effect sizes. The identified GIMs serve as candidate intermediate phenotypes in disease pathways, providing crucial insights into potential molecular pathogenic mechanisms and identifying drug targets for diseases [26]. Recently, mGWASs have been shown to play a significant role in investigating the specific metabolic pathways and regulatory mechanisms in metabolism-related diseases, such as primary hypertension [11] and coronary heart disease [12]. Among the 18 genetically influenced metabolites, androsterone sulfate, LysoPC (0:0/14:0), 16α-hydroxy DHEA 3-sulfate isomer, and L-Cystine have previously been reported [19,20,21,22]. This study identified novel mQTLs linked to these metabolites and revealed that their functional genes are closely associated with cardiovascular diseases. *EXOC4*, also known as exocyst complex component 4, serves as a regulatory gene for both androsterone sulfate and oleic acid and participates in the formation of exocyst complexes. Cullell et al. demonstrated that *EXOC4* methylation contributes to neurological changes following stroke [27]. Additionally, Levy et al. reported a link between *EXOC4* and arterial stiffness [28]. Unlike this study, other mGWASs have identified *CYP3A5* as a functional gene regulating androsterone sulfate [9,29]. This suggests that multiple genes may be involved in regulating metabolites. The functional gene for 16α-hydroxy DHEA 3-sulfate isomer, *SELPLG* (*PSGL-1*), functions as a glycoprotein that is critical for leukocyte trafficking during inflammation. *SELPLG* facilitates the homing of cytotoxic CD4 T cells to the coronary arteries, leading to plaque instability in acute coronary syndrome (ACS) [30]. Additionally, an mGWAS of 174 metabolites across six cohorts identified *GCKR* as the genetically prioritized gene for LysoPC (0:0/14:0) [21]. Yeh et al. reported that *GCKR* exon mutations exert pleiotropic effects on triglyceride and albumin levels, suggesting a potential association with the risk of cardiometabolic disorders [31].

This study identified the associations between 14 genetically influenced metabolites and their corresponding mQTLs. Among these, arabinonic acid was associated with 28 functional genes. Phosphodiesterase 3A (*PDE3A*), a member of the cGMP-inhibited cyclic nucleotide phosphodiesterase (cGI-PDE) family, primarily regulates vascular permeability, cell maturation, and phosphoric ester hydrolase activity. These processes are associated with cardiovascular diseases, including hypertension [32], stroke [33], and cardiomyopathy [34]. Ercu et al. summarized how mutations in the *PDE3A* gene contribute to hypertension and suggested that *PDE3A*-directed strategies could be utilized to treat essential hypertension and prevent hypertension-induced cardiac damage [35]. Another gene regulating arabinonic acid, *RGS6*, belongs to the RGS (G protein signaling regulator) protein family and is associated with GTPase activator activity, which plays a significant role in obesity [36] and cardiovascular disease [37]. Sengar et al. discovered that *RGS6* is involved in cardiotoxicity through its regulation of nuclein and identified *RGS6*/nuclein interaction as a potential therapeutic target for the prevention of cardiotoxicity [37]. Palmitic acid, a saturated fatty acid, has an amide derivative known as palmitic acid amide. Palmitic acid may influence cardiovascular disease risk through mechanisms beyond increasing LDL-C [38]. In a case–control study, Lee et al. found elevated palmitic amide levels in the hypertriglyceridemia (HTN) group compared to the control group, offering new insights into early metabolic changes in HTN [39]. Oleic acid, a monounsaturated fatty acid, enhances the mitochondrial oxidation of saturated fatty acids (SFA) by increasing triacylglycerol (TAG) levels and reducing diglycerol (DAG) and ceramide production, thereby protecting cells from inflammation [40]. Beneficial effects of oleic acid have been observed in hypertension [41], coronary heart disease [40], and stroke [42].

The five key genes (*CAMK2A*, *TIAM1*, *RYR2*, *RBFOX1*, and *NRXN3*) regulating GIMs were identified using the CytoHubba plug-in. These genes have been implicated in cardiovascular events, including blood pressure regulation [25,43], arrhythmias [44], and atrial fibrillation [23]. The phosphorylation of calcium/calmodulin-dependent protein kinase II (*CAMK2A*) increases the opening probability of L-type calcium channels in the sarcolemma and contributes to the development of dilated cardiomyopathy [45]. The Tiam1-Rac1 signaling module mediates the platelet-activating factor-induced disruption of interendothelial junctions and increases endothelial permeability, and it may serve as a novel therapeutic target for vascular permeability in inflammatory diseases [46]. The protein encoded by the ryanodine receptor 2 (*RYR2*), a protein component of calcium channels, regulates Ca^2+^ release from the sarcoplasmic reticulum throughout the cardiac cycle. *RYR2* mutations are associated with various arrhythmic heart diseases [44]. RNA-binding fox-1 homolog 1 (*RBFOX1*) regulates tissue-specific alternative splicing in metazoans, and its rare coding variants are associated with lower systolic blood pressure [25]. Neurexin 3 (*NRXN3*), a transmembrane adhesion protein, modulates nerve signaling and has been directly associated with SBP and DBP in GWAS [43,47]. Given the high burden of comorbidities and polypharmacy among the elderly, and the limited inclusion of this population in clinical trials, there remains a critical gap in evidence-based strategies for individualized treatment [48,49]. However, this study identified genetically influenced metabolites through the mGWAS analysis. Given the potential key roles played by these metabolites in the development of cardiovascular diseases, they may also serve as novel therapeutic targets for drug development. Future research could build upon our findings by integrating metabolite levels and genetic information to construct diagnostic or predictive models for cardiovascular diseases. Additionally, exploring metabolite-based weighted scoring systems may provide new strategies for the early identification and stratified management of salt-sensitive individuals, thereby laying a foundation for the prevention and treatment of related cardiovascular diseases.

Salt sensitivity of blood pressure is a critical risk factor for essential hypertension. Previous studies have independently explored biomarkers of SSBP through genomics and metabolomics separately [50,51]. Yang et al., using targeted metabolomics, identified L-Glutamine as a key metabolic biomarker associated with SSBP and its progression to hypertension [52]. However, single-omics approaches have limited capacity to elucidate the relationships between genes, metabolites, and SSBP. mGWASs can effectively identify associations between metabolites and genetic variation, providing crucial insights into metabolite-related genetic regulation mechanisms [53], thereby establishing a foundation for analyzing disease pathogenesis and identifying potential therapeutic targets. This study identified 14 genetically influenced metabolites with causal relationships to SSBP through MR analysis. Fumaric acid, a metabolite involved in energy metabolism, plays a significant role in salt sensitivity [54,55]. One study confirmed that the intravenous infusion of fumaric acid precursors, which elevated fumaric acid levels in the body, significantly increased the renal medullary hydrogen peroxide levels in Dahl salt-sensitive rats, causing a substantial rise in blood pressure [54]. Feruloylquinic acid, a primary source of chlorogenic acid (CGA), is linked to various health benefits, including a reduced risk of cardiovascular disease and type 2 diabetes [56]. Mills et al. conducted an acute randomized controlled crossover human intervention trial and found that CGA intake was closely correlated with flow-mediated dilatation (FMD), mediated by feruloylquinic acid in coffee [57]. Cheng et al. observed a positive correlation between L-Cystine levels and SBP or DBP. However, after adjusting for salt intake, no significant difference in L-Cystine levels was observed between the low- and high-salt-intake groups [58]. This study suggests that L-Cystine may act as a protective factor for SSBP. Therefore, further investigation is needed to determine whether L-Cystine influences SSBP.

In the replication analysis, 9 out of 18 GIMs were detectable in the replication samples, with only oleic acid and Phe-Ile showing genome-wide significant mQTL associations. This limited replication may be attributed to the stringent significance threshold and inter-batch variability in metabolite detection. Despite the small number of metabolites replicated, the consistent associations of oleic acid and Phe-Ile support their potential role in SSBP. Notably, Phe-Ile is a newly identified metabolite associated with SSBP, and current research on it remains limited. Further investigation in larger, multi-center cohorts is warranted to validate these findings and better understand their contribution to the salt sensitivity of blood pressure. Salt sensitivity, as an independent risk factor for cardiovascular events and mortality, exacerbates the global challenge of hypertension, particularly in the elderly and patients with multiple comorbidities, where the incidence of hypertension continues to rise. Genetic testing offers new possibilities for personalized hypertension management by identifying individuals at high genetic risk, enabling the development of more precise treatment strategies. However, the clinical application of genetic testing faces several challenges, such as the need for standardized diagnostic protocols and the interpretation of complex genetic data across diverse populations. Future research should focus on expanding genetic databases to include more diverse populations, further elucidating the role of identified genes in the pathogenesis of hypertension and promoting the clinical application of genetic testing in hypertension management.

This research has several strengths. Firstly, this study comprehensively identified the genetic variations associated with metabolites in human plasma and elucidated the biological mechanisms and pathways involved, thereby providing a foundation for systematically understanding the roles played by metabolites and their connections to diseases. Additionally, genetically influenced metabolites causally related to SSBP were identified, providing valuable biological insights into the early accurate identification of SSBP and the exploration of the genetic metabolic regulatory mechanisms involved in its pathogenesis. However, this study has several limitations. Firstly, the study population was limited to individuals from northern China, which potentially restricted the generalizability of these findings to other populations. Nevertheless, the results offer an important basis for understanding the genetic and metabolic regulation of SSBP in the Chinese population. Future studies should aim to validate these findings across diverse populations and research centers. Secondly, due to the lack of publicly available GWAS data for SSBP, the causal relationships between genetically influenced metabolites and SSBP identified in this study were assessed using one-sample MR. Therefore, additional public GWAS data should be sought to enable larger-scale two-sample MR analyses in the future.

## 4. Materials and Methods

### 4.1. Study Population and Samples

The Systematic Epidemiological Cohort Study of Salt Sensitivity of Blood Pressure (EpiSS) was established between 2014 and 2016 to investigate potential genetic and environmental risk factors for SSBP and their implications for cardiovascular diseases. This cohort is registered in the Chinese Clinical Trial Registry (Registration No: ChiCTR-EOC-16009980), and the detailed protocol for the cohort design has been previously published [59]. Recruitment, baseline information collection, and biological sample (blood and urine) collection for the EpiSS cohort were conducted from 2014 to 2016. SSBP was assessed using the Modified Sullivan’s Acute Oral Saline Load and Diuresis Shrinkage Test (MSAOSL-DST), with fasting venous blood samples collected before the test for subsequent analysis. In 2020, a total of 60 matched participants (30 SS vs. 30 SR) were included in this study for untargeted metabolomics and genotyping, matched by age (±5 years) and sex. The exclusion criteria included pregnancy, kidney disease, malignancies, and diabetes. After quality control (QC) of genotyping and metabolomics data, 54 participants remained for subsequent analyses. In 2024, a replication analysis and one-sample Mendelian randomization analysis were conducted, enrolling 50 SS and 50 SR subjects, selected according to the same criteria as in the discovery set. The detection of metabolites in both the discovery and replication sets was conducted using baseline blood samples. This EpiSS study adhered to the guidelines of the Declaration of Helsinki and received approval from the Ethics Committee of Capital Medical University (approval No: Z2023SY025). Written informed consent was obtained from all participants prior to their inclusion in this study.

### 4.2. Determination of SS and SR

SSBP in the EpiSS study was determined using the MSAOSL-DST method, which has been described previously [51,59]. In this test, participants ingested 1000 mL of 0.9% normal saline orally within 30 min, followed by an oral dose of 40 mg furosemide two hours after saline loading. Systolic blood pressure (SBP) and diastolic blood pressure (DBP) were measured at three time points: baseline (T_0_), 2 h after saline ingestion (T_1_), and 2 h after furosemide administration (T_2_). Blood pressure was measured three times at each time point, and the average was calculated. The mean arterial pressure (MAP) at each of the three time points (MAP_0_, MAP_1_, MAP_2_) was calculated using the following formula: MAP = (1/3 × SBP) + (2/3 × DBP). The change in MAP during the acute saline loading period (ΔMAP_1_) was defined as MAP_1_-MAP_0_, while the change during the diuresis shrinkage period (ΔMAP_2_) was defined as MAP_2_ − MAP_1_. SS individuals were defined as those with ΔMAP_1_ ≥ 5 mmHg or ΔMAP_2_ ≤ −10 mmHg, while all other participants were classified as SR individuals [59].

### 4.3. Untargeted Metabolomics Profiling and Data Processing

Metabolomics and lipidomics profiling were performed using the Ultimate 3000 ultra-high-performance liquid chromatography coupled with Q Exactive quadrupole-Orbitrap high-resolution mass spectrometer (UPLC-HRMS) system (Thermo Scientific, Waltham, MA, USA) in both positive- and negative-ion detection modes. Plasma samples were collected from the EpiSS study participants after an overnight fast and stored at −80 °C. Quality control samples were inserted at intervals of every 20 samples, and QC measures were performed to evaluate sample extraction reproducibility, instrument performance stability, and overall data quality. Compound Discoverer software (version 3.2) was used to acquire and process the metabolic data. The chemical formulas, retention time, and metabolic pathway were compared against the Human Metabolome Database (HMDB) and mzCloud online database (Thermo Scientific, Waltham, MA, USA) to annotate metabolite structures. Untargeted metabolomics and lipidomics detected and quantified a total of 970 metabolites, comprising 944 known metabolites and 26 unnamed metabolites (labeled as X).

### 4.4. DNA Extraction, Genotyping, and Quality Control

Genomic DNA was extracted and quantified from peripheral blood samples using the Magnetic Bead Whole Blood Genomic DNA Extraction Kit (BioTeke, Beijing, China) according to standard quality control procedures. Genomic DNA samples from 54 participants were genotyped using the Illumina Infinium Asian Screening Array BeadChip-24 v1.0 (ASA, Illumina, San Diego, CA, USA). Quality control was performed based on the following criteria: (1) SNP loci with a call rate < 95% were eliminated; (2) SNP loci with a minor allele frequency (MAF) < 0.01 were removed; (3) SNP loci with Hardy–Weinberg equilibrium (*p*-value < 1 × 10^−4^) in the control group were excluded; (4) sex chromosomes were filtered; (5) samples with a call rate < 95% were excluded; (6) samples with an abnormal heterozygosity rate > 6 standard deviations were excluded; (7) and samples with PI-HAT (kinship) > 0.25 were excluded.

After quality control, 54 samples and 483,002 SNP loci remained. GWAS data imputation was conducted using IMPUTE2 software (version 2.3.1), with the Genome Reference Consortium Human Genome Build 37 (GRCh37) as the reference (https://www.ncbi.nlm.nih.gov/datasets/genome/GCF_000001405.13/, accessed on 23 March 2024). SNP loci with INFO > 0.8, MAF > 0.01, and a call rate > 0.95, were retained, resulting in 54 samples and 4,241,225 loci for subsequent analysis.

### 4.5. mGWAS Analysis

In this study, an mGWAS of 970 untargeted metabolites was conducted using PLINK software (version 1.09). Correlation analysis was conducted under the additive genetic model using linear regression, adjusting for age, sex, BMI, and the first five principal components. To control the false-positive rate of multiple comparisons, the statistical significance threshold for associations between SNPs and metabolites was set at a Bonferroni-adjusted *p*-value of *p* < 5 × 10^−8^/970 = 5.15 × 10^−11^. The R software package “CMplot” was used to generate the Manhattan plot visualizing the mGWAS result.

Linkage disequilibrium was assessed using PLINK software with the threshold of *r*^2^ < 0.02 and a window size of 500 kb. Gene mapping and annotation for each independent mQTL were performed using the PLINK software (version 1.09). The mQTL gene annotation results were visualized with the PhenoGram online tool (http://visualization.ritchielab.org/, accessed on 14 September 2024). The biological processes, molecular functions, and pathways associated with mQTL-related genes were explored using Gene Ontology (GO) enrichment analysis and Kyoto Encyclopedia of Genes and Genomes (KEGG) pathway analysis. Protein–protein interaction (PPI) network information was retrieved from STRING 12.0 and was visualized using Cytoscape 3.10.0 software. The top five key genes were identified using the maximal clique centrality (MCC) algorithm, performed using the CytoHubba plug-in of Cytoscape.

### 4.6. Statistical Analysis

All statistical analyses were conducted using R software (version 4.3.3). Continuous variables were expressed as mean ± standard deviation (SD), and categorical variables were described as frequency (percentage). Student’s *t*-test was used to compare normally distributed continuous variables between the two groups, whereas the Mann–Whitney U-test was employed to analyze non-normally distributed and ranked variables. Differences in categorical variables between groups were assessed using the Chi-square test or Fisher’s exact test. A two-sided *p*-value < 0.05 was considered statistically significant.

The multivariable logistic regression model was constructed to assess the association between GIMs and SSBP, adjusting for age, sex, and the first five principal components. One-sample MR methods were employed to infer potential causal relationships between genetically influenced metabolites and SSBP. Unweighted genetic risk scores (uGRSs) and weighted genetic risk scores (wGRSs) were constructed for each GIM as instrumental variables. The two-stage least squares (2SLSs) method was used to analyze the causal relationship between GIMs and SSBP. The 2SLSs method comprised two stages. In the first stage, the association strength between GRS and GIMs was assessed using linear regression to estimate the predicted value of the GIMs. In the second stage, the association between the predicted values from the first stage and SSBP was analyzed using logistic regression, adjusted for age and gender. The odds ratios (ORs) and 95% confidence interval (95% CI) were used to quantify the strength of the causal association between GIMs and SSBP. All statistical analyses were conducted using R software (version 4.3.3).

### 4.7. Replication Set

Serum metabolomics analysis was conducted on 100 participants in the replication set. The genetically influenced metabolites identified in the discovery set were then assessed through the mGWAS analysis. One-sample MR analyses were performed for the GIMs with significant mQTLs to conduct replication analysis and assess the reliability of the findings.

## 5. Conclusions

This study utilized the mGWAS of plasma metabolites to identify associations between 18 genetically influenced metabolites and 368 independent mQTLs. We identified key regulatory genes influencing genetically influenced metabolites. The associated genes are primarily involved in the signal transduction of sinoatrial node cells, the regulation of transmembrane receptor protein kinase activity, and cellular metabolism. One-sample MR analysis identified 14 genetically influenced metabolites with a causal relationship with SSBP. The replication analysis showed that the genetically influenced metabolite Phe-lle remained causally associated with SSBP. The findings of this study provide a crucial foundation for understanding the functional regulations between genetics and metabolites, as well as for exploring the potential drug targets and pathogenic mechanisms underlying SSBP.

## Figures and Tables

**Table 1:** Characteristics of participants and comparisons between salt-sensitive and salt-resistant groups.

Variables	Total (*n* = 54)	SS (*n* = 30)	SR (*n* = 24)	*p*
Age (year)	57.57 ± 5.43	57.73 ± 5.77	57.38 ± 5.11	0.812 ^a^
Gender (Male, *n*, %)	17 (31.5)	8 (33.3)	9 (30.0)	1.000 ^c^
BMI (kg/m^2^)	26.62 ± 3.52	26.39 ± 3.26	26.92 ± 3.86	0.586 ^a^
FBG (mmol/L)	5.45 ± 0.63	5.44 ± 0.61	5.47 ± 0.66	0.924 ^b^
TC (mmol/L)	5.48 ± 0.94	5.37 ± 1.03	5.61 ± 0.80	0.360 ^a^
TGs (mmol/L)	1.83 ± 1.03	1.86 ± 1.09	1.79 ± 0.96	0.721 ^b^
LDL-C (mmol/L)	2.32 ± 0.91	2.24 ± 0.88	2.42 ± 0.95	0.461 ^a^
HDL-C (mmol/L)	1.95 ± 1.17	1.93 ± 1.21	1.97 ± 1.14	0.614 ^b^
SBP (mmHg)	122.01 ± 20.45	114.19 ± 21.10	131.78 ± 14.94	0.001 ^a^
DBP (mmHg)	78.11 ± 12.02	71.72 ± 10.72	86.10 ± 8.25	<0.001 ^a^
MAP (mmHg)	92.74 ± 13.39	85.87 ± 12.52	101.33 ± 8.78	<0.001 ^a^
ΔMAP_1_ (mmHg)	−2.62 ± 15.14	10.16 ± 3.31	−18.59 ± 5.92	<0.001 ^b^
ΔMAP_2_ (mmHg)	−2.07 ± 10.78	−10.48 ± 5.44	8.44 ± 4.81	<0.001 ^a^
Smoking (*n*, %)	7 (13.0)	5 (16.7)	2 (8.3)	0.443 ^d^
Drinking (*n*, %)	26 (48.1)	16 (53.3)	10 (41.7)	0.563 ^c^
Hypertension (*n*, %)	28 (51.9)	15 (50.0)	13 (54.2)	0.976 ^c^
Family history of hypertension (*n*, %)	29 (53.7)	18 (60.0)	11 (45.8)	0.446 ^c^

Abbreviations: SS, salt-sensitive; SR, salt-resistant; BMI, body mass index; FBG, fasting blood glucose; TC, total cholesterol; TGs, triglycerides; LDL-C, low-density lipoprotein cholesterol; HDL-C, high-density lipoprotein cholesterol; SBP, systolic blood pressure; DBP, diastolic blood pressure; MAP, mean arterial pressure; ΔMAP_1_, mean arterial pressure change during the acute saline load period; ΔMAP_2_, mean arterial pressure change during the diuresis shrinkage period. ^a^, Student’s *t*-test; ^b^, Mann–Whitney U-test; ^c^, statistical analysis by the χ^2^ test; ^d^, Fisher’s exact test.

**Table 2:** Linkage disequilibrium analysis identified independent mQTLs for 18 GIMs.

GIMs	Numbers of mQTLs	SNP *	CHR	Position	Minor Allele	Other Allele	MAF	*p*
Arabinonic acid	67	rs3792688	14	36788896	G	C	0.010	7.13 × 10^−21^
Feruloylquinic acid	46	rs11076702	16	88774569	A	C	0.019	4.20 × 10^−15^
Phe-lle	43	rs11758014	6	148830586	C	G	0.010	2.73 × 10^−16^
Phe-Phe	42	rs111865319	14	23507554	T	G	0.010	4.08 × 10^−16^
LysoPC (0:0/22:5*n*-3)	34	rs17773637	2	103168041	A	T	0.010	1.40 × 10^−13^
Androsterone sulfate	29	rs193142735	7	28077870	T	A	0.010	1.34 × 10^−13^
Fumaric acid	29	rs117266991	6	93172013	G	A	0.028	3.96 × 10^−14^
TGs (12:0/12:0/12:0)	24	rs11076702	16	88774569	A	C	0.019	4.20 × 10^−15^
Glycoursodeoxycholate 3-glucuronide	23	rs111941908	2	29660776	G	A	0.019	9.95 × 10^−14^
Oleic acid	19	rs148784825	1	196024278	C	T	0.019	4.41 × 10^−15^
LysoPC (0:0/14:0)	2	rs7523503	1	200899804	A	G	0.151	4.60 × 10^−11^
16α-hydroxy DHEA 3-sulfate isomer	2	rs3782520	12	109027340	T	C	0.074	1.25 × 10^−11^
L-Cystine	2	rs17812386	9	113942888	A	G	0.029	1.65 × 10^−13^
X-MZ140RT42	2	rs117266991	6	93172013	G	A	0.028	3.16 × 10^−12^
Glycoursocholic acid	1	rs117458538	16	86932146	G	A	0.056	6.90 × 10^−12^
DG (18:0/18:0)	1	rs146258959	18	34962385	C	T	0.028	2.40 × 10^−11^
Palmitic amide	1	rs118008665	17	59640888	T	C	0.028	5.92 × 10^−12^
*N*-Acetyl-l-aspartic acid	1	rs145741649	5	105503858	A	G	0.020	2.89 × 10^−11^

Abbreviations: *, only the top SNP is listed for each metabolite; GIMs, genetically influenced metabolites; numbers of mQTLs, number of mQTLs retained after linkage disequilibrium analysis for each metabolite; CHR, chromosome; SNP, single nucleotide polymorphism; MAF, minor allele frequency.

**Table 3:** One-sample mendelian randomization analysis of genetically influenced metabolites and SSBP.

GIMs	Model 1 ^a^			Model 2 ^b^		
	*β*	ORs (95%CI)	*p*	*β*	ORs (95%CI)	*p*
Arabinonic acid	−0.087	0.916 (0.866, 0.970)	0.004	−0.085	0.919 (0.871, 0.970)	0.003
Feruloylquinic acid	−0.052	0.949 (0.915, 0.984)	0.007	−0.054	0.947 (0.915, 0.981)	0.003
Phe-lle	−0.097	0.908 (0.881, 0.936)	<0.001	−0.093	0.911 (0.887, 0.935)	<0.001
Phe-Phe	−0.088	0.915 (0.892, 0.939)	<0.001	−0.087	0.916 (0.894, 0.939)	<0.001
LysoPC (0:0/22:5*n*-3)	0.092	1.097 (1.046, 1.150)	<0.001	0.093	1.098 (1.047, 1.151)	<0.001
Androsterone sulfate	−0.065	0.937 (0.909, 0.967)	<0.001	−0.066	0.937 (0.908, 0.966)	<0.001
Fumaric acid	−0.563	0.570 (0.389, 0.834)	0.006	−0.564	0.569 (0.388, 0.834)	0.006
TGs (12:0/12:0/12:0)	−0.182	0.833 (0.794, 0.875)	<0.001	−0.180	0.835 (0.796, 0.877)	<0.001
Glycoursodeoxycholate 3-glucuronide	−0.053	0.948 (0.928, 0.969)	<0.001	−0.056	0.946 (0.926, 0.966)	<0.001
Oleic acid	−0.044	0.957 (0.934, 0.981)	0.001	−0.045	0.956 (0.934, 0.979)	<0.001
LysoPC (0:0/14:0)	−0.353	0.702 (0.553, 0.891)	0.005	−0.353	0.702 (0.553, 0.891)	0.005
16α-hydroxy DHEA 3-sulfate isomer	−0.037	0.964 (0.881, 1.055)	0.428	−0.027	0.973 (0.887, 1.068)	0.567
L-Cystine	−0.207	0.813 (0.711, 0.930)	0.004	−0.198	0.820 (0.726, 0.927)	0.002
X-MZ140RT42	−0.528	0.590 (0.428, 0.814)	0.002	−0.533	0.587 (0.423, 0.815)	0.002
Glycoursocholic acid	−0.050	0.951 (0.895, 1.011)	0.117	−0.050	0.951 (0.895, 1.011)	0.117
DG (18:0/18:0)	0.068	1.071 (0.798, 1.437)	0.652	0.068	1.071 (0.798, 1.437)	0.652
Palmitic amide	0.140	1.150 (0.673, 1.965)	0.611	0.140	1.150 (0.673, 1.965)	0.611
*N*-Acetyl-l-aspartic acid	−0.486	0.615 (0.466, 0.811)	0.001	−0.486	0.615 (0.466, 0.811)	0.001

Abbreviations: ^a^, two-stage least squares analysis of the unweighted genetic risk score of metabolites and SSBP; ^b^, two-stage least squares analysis of the weighted genetic risk score of metabolites and SSBP. The *p*-value was calculated using the 2SLSs method, adjusted for age and gender.

## Data Availability

The original contributions presented in this study are included in the article/Supplementary Materials. Further inquiries can be directed to the corresponding author.

## Supplementary Materials

The following supporting information can be downloaded at: https://www.mdpi.com/article/10.3390/ijms26104538/s1.

## Abbreviations

The following abbreviations are used in this manuscript:

GIM	Genetically influenced metabolite
mGWAS	Metabolome genome-wide association study
MR	Mendelian randomization
LD	Linear dichroism
SSBP	Salt sensitivity of blood pressure
SS	Salt-sensitive
SR	Salt-resistant
GWAS	Genome-wide association study
mQTLs	Metabolic quantitative trait loci
SBP	Systolic blood pressure
DBP	Diastolic blood pressure
MAP	Mean arterial pressure
uGRSs	Unweighted genetic risk scores
wGRSs	Weighted genetic risk scores
2SLSs	Two-stage least squares
