# Supplementary figures and images for: Novel Metabolites Genetically Linked to Salt Sensitivity of Blood Pressure: Evidence from mGWAS in Chinese Population
Source: Int J Mol Sci. 2025 May 9;26(10):4538. doi: 10.3390/ijms26104538 (PMC12111247; doi:10.3390/ijms26104538)

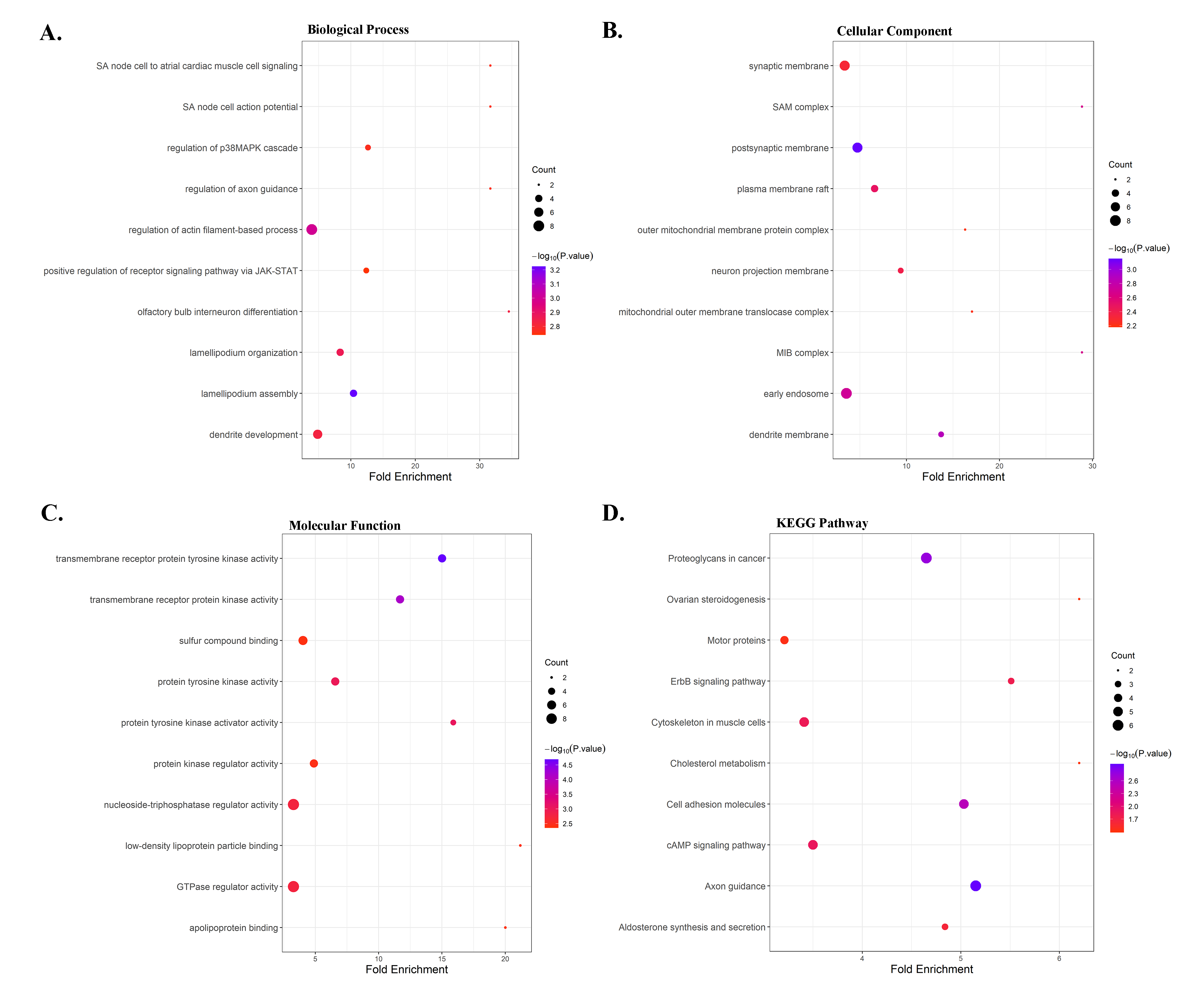

Supplement: Supplementary file 1 [file ijms-26-04538-s001.zip › Supplementary Figure 1.tif]
